# Are Oligotypes Meaningful Ecological and Phylogenetic Units? A Case Study of *Microcystis* in Freshwater Lakes

**DOI:** 10.3389/fmicb.2017.00365

**Published:** 2017-03-08

**Authors:** Michelle A. Berry, Jeffrey D. White, Timothy W. Davis, Sunit Jain, Thomas H. Johengen, Gregory J. Dick, Orlando Sarnelle, Vincent J. Denef

**Affiliations:** ^1^Department of Ecology and Evolutionary Biology, University of MichiganAnn Arbor, MI, USA; ^2^Department of Biology, Framingham State UniversityFramingham, MA, USA; ^3^NOAA Great Lakes Environmental Research LaboratoryAnn Arbor, MI, USA; ^4^Department of Earth and Environmental Sciences, University of MichiganAnn Arbor, MI, USA; ^5^Cooperative Institute for Limnology and Ecosystems Research, University of MichiganAnn Arbor, MI, USA; ^6^Department of Fisheries and Wildlife, Michigan State UniversityEast Lansing, MI, USA

**Keywords:** oligotypes, microbial species, ecotypes, dada2, 16S rRNA gene sequencing, *Microcystis*

## Abstract

Oligotyping is a computational method used to increase the resolution of marker gene microbiome studies. Although oligotyping can distinguish highly similar sequence variants, the resulting units are not necessarily phylogenetically and ecologically informative due to limitations of the selected marker gene. In this perspective, we examine how oligotyping data is interpreted in recent literature, and we illustrate some of the method’s constraints with a case study of the harmful bloom-forming cyanobacterium *Microcystis*. We identified three *Microcystis* oligotypes from a western Lake Erie bacterial community 16S rRNA gene (V4 region) survey that had previously clustered into one OTU. We found the same three oligotypes and two additional sequence variants in 46 *Microcystis* cultures isolated from Michigan inland lakes spanning a trophic gradient. In Lake Erie, shifts in *Microcystis* oligotypes corresponded to spatial nutrient gradients and temporal transitions in bloom toxicity. In the cultures, *Microcystis* oligotypes showed preferential distributions for different trophic states, but genomic data revealed that the oligotypes identified in Lake Erie did not correspond to toxin gene presence. Thus, oligotypes could not be used for inferring toxic ecotypes. Most strikingly, *Microcystis* oligotypes were not monophyletic. Our study supports the utility of oligotyping for distinguishing sequence types along certain ecological features, while it stresses that 16S rRNA gene sequence types may not reflect ecologically or phylogenetically cohesive populations. Therefore, we recommend that studies employing oligotyping or related tools consider these caveats during data interpretation.

## Interpretation of Oligotyping Patterns Hinges on Mostly Untested Assumptions Regarding the Ecological and Phylogenetic Cohesion of Oligotypes

Microbiome studies using 16S rRNA gene amplicons typically aggregate sequences into operational taxonomic units (OTUs) based on a 97% sequence identity threshold. The OTU approach is used, in part, to mitigate effects of high error rates from high-throughput sequencing technologies. However, OTU methods throw out potentially informative 16S sequence variation and can group together ecologically distinct populations (Coleman et al., 2006; Hunt et al., 2008; Denef et al., 2010; Shapiro and Polz, 2014).

As an alternative to OTUs, the oligotyping method can distinguish real sequence variants from sequencing errors, and can segregate sequence types that differ by as a little as a single nucleotide (Eren et al., 2013). The increased resolution offered by this approach allegedly enhances the likelihood of identifying ecotypes. For example, *Pelagibacter* oligotypes from a coastal marine environment alternated in dominance during low and high temperature periods of the year, suggesting that the oligotypes occupied separate niches (Eren et al., 2013). Due to the potential increase in ecological resolution, the oligotyping method has been broadly applied for studies in microbial biogeography (Schmidt et al., 2014; Buttigieg and Ramette, 2015; Cloutier et al., 2015; Newton and McLellan, 2015), host–microbe associations (Eren et al., 2014; Menke et al., 2014; Fisher et al., 2015), and links between microbes and disease (Eren et al., 2011).

However, to conclude that oligotypes represent ecotypes, one must consider assumptions about the ecological (shared traits) and evolutionary cohesiveness (derived from a single common ancestor, distinct from other ecotypes) of ‘populations’ defined by fine-scale nucleotide variation in 16S rRNA gene hypervariable regions. The meaning of a microbial species or ecotype is still highly debated, but experimental and theoretical work has converged on a definition that includes inhabiting the same ecological niche, exhibiting constrained genetic diversity, and belonging to a distinct evolutionary lineage (Gevers et al., 2005; Cohan and Perry, 2007; Koeppel et al., 2008). Exercising caution when interpreting oligotyping results is warranted, because the 16S rRNA gene, even at full length, can miss important genetic variation underlying ecological and evolutionary differentiation between species (Jaspers and Overmann, 2004; Konstantinidis and Tiedje, 2005; Maiden et al., 2013; Kim et al., 2014; Hahn et al., 2016). From an ecological perspective, many bacterial functional traits are not phylogenetically conserved and are therefore unlikely to be predicted from the 16S rRNA gene (Martiny et al., 2013). From an evolutionary perspective, the 16S rRNA gene is a slowly evolving gene (Ochman et al., 1999), that while useful for assigning high level bacterial taxonomy, may not resolve more recent evolutionary diversification within a lineage.

Despite references in many articles (e.g., Delmont et al., 2014; Schmidt et al., 2014; Kleindienst et al., 2015), the hypothesis that 16S rRNA gene oligotypes represent ecotypes or species-like groups is largely untested. The outcome of this hypothesis has broad implications for other inferential claims found in the literature regarding co-evolution, e.g., between animal hosts and their associated microbes (e.g., Menke et al., 2014), or biogeography of microbes (e.g., Schmidt et al., 2014; Buttigieg and Ramette, 2015; Cloutier et al., 2015; Newton and McLellan, 2015). Some studies have already emphasized the need to conduct studies beyond marker gene analysis that can test the ecological hypotheses generated from oligotyping surveys (e.g., Eren et al., 2013; Buttigieg and Ramette, 2015). We propose to examine these assumptions using two tools designed to differentiate closely related taxa: by performing multi-locus sequence typing (MLST) (Maiden et al., 2013), which aims to reveal more resolved phylogenetic relationships based on a set of neutrally evolving housekeeping genes, and by targeting genes that underpin functional traits (in our case toxin production).

It is an important time to validate methods for improving resolution of 16S rRNA gene surveys, because the number of available methods is increasing. Minimum Entropy Decomposition (MED) is a high-throughput extension of oligotyping that can be applied to whole microbial community datasets (Eren et al., 2015), and otu2ot provides an R software interface to the oligotyping and MED methods (Ramette and Buttigieg, 2014). In addition, the recently developed dada2 tool also aims to increase resolution of marker gene surveys, but does so by explicitly modeling transition rates between closely related sequences (Callahan et al., 2016). The points we raise in this perspective apply equally to the interpretation of oligotyping, MED, dada2, and any other tools attempting to make ecotype inferences from 16S rRNA gene amplicons.

## Microcystis Case Study

To illustrate potential issues with the ecological and evolutionary assumptions made about 16S rRNA oligotypes, we present a case study of *Microcystis*, a colony-forming cyanobacterium that is a prominent component of harmful algal blooms in freshwater systems worldwide (Harke et al., 2016b; O’Neil et al., 2012). First, we oligotyped *Microcystis* reads from a bacterial community dataset sampled over three sites and 20 weeks from western Lake Erie during the 2014 cyanobacterial harmful algal bloom. We analyzed oligotypes with respect to two parameters hypothesized to reflect key ecological traits: concentration of microcystin (toxin potential) and total phosphorus (trophic preference). This approach is comparable to the setup of previously published oligotyping studies, in that we attempted to link uncharacterized oligotypes from a community dataset to environmental gradients. Next, we examined oligotypes and genomes from 46 *Microcystis* cultures (oligotypes were determined after combining the Lake Erie and the isolate sequence data), which were isolated (together with their colony-associated heterotrophic bacteria) from 14 Michigan inland lakes in 2011 and 2013. Comparing oligotypes from the Lake Erie community samples with the cultures provided multiple advantages. The cultures were isolated from single *Microcystis* colonies and were typically of clonal origin, which served to constrain the considered population. In addition, the culture collection allowed us to compare, with high accuracy, the gene content of each *Microcystis* isolate with its oligotype, and to construct an MLST phylogeny based on five housekeeping genes.

**FIGURE 1:**
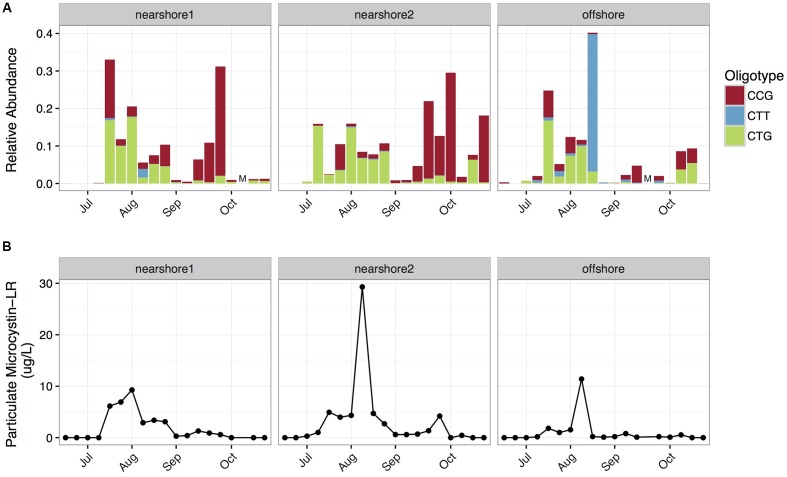
**Spatiotemporal distribution of *Microcystis* oligotypes in western Lake Erie. (A)** The relative abundance of *Microcystis* oligotypes, as fraction of total bacterial reads, from three sites in Western Lake Erie over time. The offshore site had lower median total phosphorus and chlorophyll a levels than the two nearshore sites. Samples were taken from the retentate of 2 L lake water filtered through a 100 μm filter. M denotes missing samples. **(B)** Particulate Microcystin-LR concentrations over sites and time.

*Microcystis* was the dominant large-colony forming cyanobacterial genus in the 2014 cyanobacterial harmful algal bloom in western Lake Erie (Berry et al., 2016). Analysis with mothur produced a single abundant *Microcystis* OTU, while oligotyping produced subdivisions of the OTU into three sequence variants (CTG, CCG, CTT; Supplementary Figure S1), which exhibited differing spatial and temporal dynamics (**Figure 1**). We observed that the CTG variant dominated in July and August (median CTG:CCG ratio = 4.3), but the CCG variant dominated in September and October (median CTG:CCG ratio = 0.23). The transition between these two sequence variants coincided with a shift in bloom toxicity from high to low (**Figure 1**), a trend that has been documented in other bloom years on Lake Erie (Gobler et al., 2016). We hypothesized that CTG might represent a toxic ecotype, and CCG might represent a non-toxic ecotype. Indeed, the relative abundance of CTG was positively correlated with particulate microcystin-LR levels (Spearman’s rho: 0.71, *p* < 0.001). However, it’s important to note that this correlation may be unstable due to the non-stationarity of the data (see details in Supplementary Methods Section).

The maximum and median relative abundance of CTT was an order of magnitude higher at the offshore station (maximum = 37.0%; median = 0.28%) than the nearshore stations (maximum = 2.3%, median = 0.023%). Since the offshore station had lower median phosphorus levels (Supplementary Table S1), we hypothesized that the oligotypes might underlie differences in competitive abilities along trophic gradients.

**FIGURE 2:**
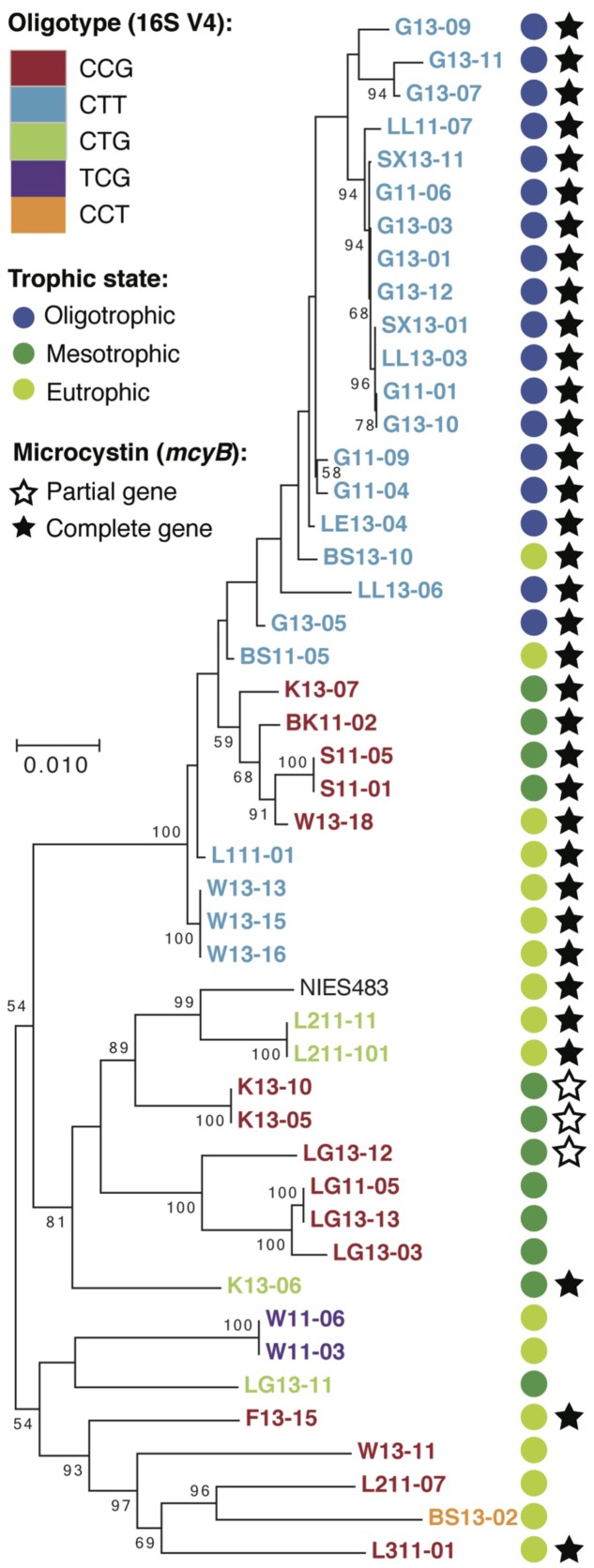
**RAxML tree for cultured *Microcystis* strains based on five concatenated housekeeping genes (pgi, gltX, ftsZ, glnA, gyrB)**. Presence of microcystin biosynthesis gene was determined from assembly and retrieval of *Microcystis* genes from the *Microcystis*-heterotroph co-culture metagenome. Trophic status of the lake was determined from total phosphorus levels (Supplementary Tables S1, S2).

Next, we examined 16S rRNA gene and whole genome data from the collection of *Microcystis* isolate cultures (Supplementary Table S2) to further investigate our hypotheses about *Microcystis* oligotypes, toxicity, and trophic status. Similar to the Lake Erie dataset, all *Microcystis* 16S rRNA gene V4 region sequences clustered into one OTU, but we recovered five oligotypes (CTG, CCG, CTT, TCG, CCT). Three of these matched the oligotypes found in Lake Erie. Although the oligotypes derived from the cultures did not indicate the trophic status of the inland lakes, the trophic status could predict which oligotypes were present (**Figure 2**). For example, CTT was the only oligotype present in oligotrophic lakes, CCG and CTG were the only oligotypes present in mesotrophic lakes, but all five oligotypes were present in inland eutrophic lakes. These data support that fine-scale variation in the 16S rRNA gene V4 region might distinguish populations with differing competitive abilities along nutrient gradients. Specifically, CTT might exclude other oligotypes from oligotrophic environments, yet all three oligotypes might coexist in the eutrophic environments due to intra-lake spatial or temporal variation in nutrient concentrations. Recent work from western Lake Erie demonstrates that *Microcystis* populations upregulate phosphorus scavenging genes in response to low phosphorus conditions at offshore sites, leading to a competitive advantage over other cyanobacterial taxa (Harke et al., 2016a). Our data suggest that low phosphorus conditions could also select for particular *Microcystis* oligotypes. Similar to hypotheses by other authors based on oligotyping/MED analyses, this hypothesis will need to be more formally tested.

A second hypothesis derived from our Lake Erie observations was that *Microcystis* oligotypes represent ecotypes that differ in their ability to produce toxins. However, in the cultures the oligotypes did not unequivocally correspond to the presence of genes for microcystin biosynthesis. Therefore, despite a correlation between oligotypes and toxicity in the Lake Erie dataset, we could not corroborate that the CTG variant can generally be assumed to be predictive of a toxic genotype and the CCG variant predictive of a non-toxic genotype. These data are consistent with previous reports that strains containing the toxin producing *mcy* gene cluster form a polyphyletic group in *Microcystis* and other toxin-producing *Cyanobacteria* (Otsuka et al., 1999; Kurmayer et al., 2014). Furthermore, a recent review of global *Microcystis* diversity indicates that 27 strains, varying in toxic potential, exhibit 99.4–99.93% similarity across the full length 16S rRNA gene (Harke et al., 2016b), so surveys based on shortened 16S rRNA gene amplicons are likely to group several toxic and non-toxic populations together. Other studies have used loci, such as the internal transcribed spacer (ITS), to provide higher genetic resolution of *Microcystis* strains (Bozarth et al., 2010; Lemaire et al., 2012; Pobel et al., 2012). These studies have produced variable results with regards to identifying associations between sequence types and environmental or biotic parameters. Thus, for any study, it is necessary to choose a suitable gene marker for the ecological trait of interest. When the distribution of the ecological trait is unknown, correlations between gene markers and environmental gradients should be interpreted with due caution.

Although our comparison of environmental community samples with cultures provided distinct advantages for implementing MLST and gene targeting analyses, this approach also has some limitations. The cultures were not derived from Lake Erie, so the cultures may or may not represent the same populations found in the community dataset. However, our analysis does not hinge on this assumption. The community dataset demonstrates that it is easy to correlate oligotypes with a trait or environmental gradient, while the cultures demonstrate that variation in the 16S gene is not a robust proxy for the traits we considered. Importantly, we argue that the point of defining ecotypes using sequencing based methods is to be able to link ecological traits to sequence types with predictive power to other environments. Hence, it was appropriate to combine the two datasets.

As for the evolutionary interpretation of oligotypes, an MLST analysis performed on the culture genomic data revealed two main results. First, strains of the same oligotype did not always form monophyletic groups (**Figure 2**). While bootstrap support values were low for several of the branching points, in part due to high sequence similarity of the MLST genes within the isolate collection, several instances of polyphyletic oligotype groups were nonetheless evident. Second, the number of nucleotide differences in the 16S rRNA gene V4 region was not consistent with MLST-based patristic distances (Supplementary Figure S2). For example, oligotypes with two or three nucleotide differences in the 16S rRNA gene V4 region were not more distant on the MLST tree than oligotypes differing by one nucleotide. This indicated that *Microcystis* V4 oligotypes were not phylogenetically cohesive units. Previous work has shown that, surprisingly, many OTUs across bacterial phyla are not monophyletic (Koeppel and Wu, 2013). Our data support this observation and demonstrate the principle that 16S rRNA gene hypervariable regions can be poor proxies for evolutionary distance at finer taxonomic scales, irrespective of whether the resolution is at the OTU or oligotype level.

In summary, distributions of *Microcystis* oligotypes from an environmental community dataset corresponded with shifts in toxicity and spatial variation in phosphorus levels. However, an additional analysis leveraging genomic data from *Microcystis* cultures revealed that strains with the same oligotype (including oligotypes corresponding to those detected in Lake Erie) varied in whether the toxin gene was present or not. In addition, isolates belonging to the same oligotypes did not consistently form monophyletic groups. Thus *Microcystis* 16S rRNA gene amplicons may be useful to discriminate ecologically distinct populations when more complex and presumably multi-genic traits are considered, such as shifts on the oligotroph-copiotroph spectrum (Lauro et al., 2009; Martiny et al., 2013). However, when we focus on ecological traits that are underpinned by a single or a handful of genes in the flexible genome, such as toxin production, 16S rRNA gene amplicons may carry limited information and single nucleotide variants may lead us to unwarranted conclusions.

As this study focused on only one organismal group, we cannot determine how broadly applicable our results are to other studies. For example, other organisms may exhibit a closer correlation between fine-scale variation in 16S rRNA hypervariable regions and phylogenetic distance. Still, we aim to highlight that phylogenetic cohesiveness is a critical requirement to be able to equate oligotypes to ecotypes and should be considered in any marker gene study using oligotyping methods. In addition, the relevance of oligotypes for predicting ecotypes is largely dependent on the marker gene used and the ecological trait considered in each study. In some cases, the selected marker gene may be a reasonable proxy for ecological groups – trophic preference of *Microcystis* 16S rRNA gene oligotypes may represent one such case. However, even for trophic state, which corresponds to the relatively large environmental gradients often considered in previous oligotyping surveys, the position along the gradient predicted which oligotypes were present, but the reverse was not true. This again indicates that one oligotype may represent multiple ecotypes. As such, we caution any study that aims to draw ecological inferences based purely on correlations between oligotypes and environmental gradients (e.g., Kleindienst et al., 2015). While we are limited to this specific case study, we hope it will prompt others to more broadly investigate lack of trait conservation and polyphyly within oligotypes.

## Recommendations for Use of Oligotyping, MED, and Dada2

Oligotyping, MED, and dada2 provide higher sequence resolution to marker gene surveys, but they are inherently constrained by the resolving power of the gene considered. In the case of the 16S rRNA gene, which has been the marker gene used thus far, it is well known that it has limited ability to discriminate species/ecotype level groups (Jaspers and Overmann, 2004; Konstantinidis and Tiedje, 2005; Maiden et al., 2013; Kim et al., 2014; Hahn et al., 2016). Our data confirm this previously reported property by showing (1) limited predictive power from oligotypes to traits, and (2) limited predictive power from oligotypes to MLST-based subclades. Importantly, oligotyping methods can be applied to any gene, though care must be taken to consider selection pressures acting upon the chosen genes. In both the experimental design and data interpretation stages, researchers should carefully consider which marker gene to use for their survey and whether observed correlations to environmental gradients can be biologically supported by additional empirical evidence.

The use of oligotyping, MED, and dada2 for 16S rRNA gene surveys remains useful because it maximizes the potential sequence type resolution from high-throughput sequencing studies that can then be used to formulate ecological hypotheses. However, even at its maximum resolution, i.e., full-length high quality sequences, the 16S rRNA has limited sensitivity to resolve ecological and evolutionary variation between closely related lineages. As the application of these tools is likely to rapidly increase in the coming years, we reiterate the original authors’ statements that these methods should be used only as a foundation to generate ecological hypotheses from microbial community datasets (Eren et al., 2013). The increased resolution enabled by these new methods should not preclude rigorous use of ecological and evolutionary terms and concepts.

## Data Access

Sequences used for oligotyping analyses are available under SRA accession numbers PRJNA318386 (Lake Erie 16S rRNA gene; Supplementary Table S3), and PRJNA351875 (inland lakes 16S rRNA gene and metagenomic data; Supplementary Table S2). Sequences used for MLST analyses are available under GenBank accession numbers KY009968 – KY010196. All sample data and code to fully reproduce analyses is available at https://github.com/DenefLab/microcystis-oligotypes (hash 3c2bfb1, December 22, 2016) and as Supplementary Code to this publication.

## Author Contributions

MB, JW, OS, TD, TJ, GD, and VD designed the experiment. JW, MB, TD, and TJ collected data. MB, SJ, and VD analyzed data. MB and VD wrote the paper.

## Conflict of Interest Statement

The authors declare that the research was conducted in the absence of any commercial or financial relationships that could be construed as a potential conflict of interest.

## References

[B1] Berry M. A. Davis T. W. Cory R. M. Duhaime M. B. Johengen T. H. Kling G. W. (2016). Cyanobacterial harmful algal blooms are a biological disturbance to western Lake Erie bacterial communities. *Environ. Microbiol.* 10.1111/1462-2920.13640 [Epub ahead of print]. 28026093

[B2] Bozarth C. S. Schwartz A. D. Shepardson J. W. Colwell F. S. Dreher T. W. (2010). Population turnover in a *Microcystis* bloom results in predominantly nontoxigenic variants late in the season. *Appl. Environ. Microbiol.* 76 5207–5213. 10.1128/AEM.00001-10 20543038 PMC2916505

[B3] Buttigieg P. L. Ramette A. (2015). Biogeographic patterns of bacterial microdiversity in Arctic deep-sea sediments (Hausgarten, Fram Strait). *Front. Microbiol.* 6:660 10.3389/fmicb.2014.00660 PMC4283448 25601856

[B4] Callahan B. J. Mcmurdie P. J. Rosen M. J. Han A. W. Johnson A. J. A. Holmes S. P. (2016). DADA2: high-resolution sample inference from Illumina amplicon data. *Nat. Methods* 13 581–583. 10.1038/nmeth.3869 27214047 PMC4927377

[B5] Cloutier D. D. Alm E. W. McLellan S. L. (2015). Influence of land use, nutrients, and geography on microbial communities and fecal indicator abundance at Lake Michigan beaches. *Appl. Environ. Microbiol.* 81 4904–4913. 10.1128/AEM.00233-15 25979888 PMC4495187

[B6] Cohan F. M. Perry E. B. (2007). A systematics for discovering the fundamental units of bacterial diversity. *Curr. Biol.* 17 R373–R386. 10.1016/j.cub.2007.03.032 17502094

[B7] Coleman M. L. Sullivan M. B. Martiny A. C. Steglich C. Barry K. Delong E. F. (2006). Genomic islands and the ecology and evolution of *Prochlorococcus*. *Science* 311 1768–1770. 10.1126/science.1122050 16556843

[B8] Delmont T. O. Hammar K. M. Ducklow H. W. Yager P. L. Post A. F. (2014). *Phaeocystis antarctica* blooms strongly influence bacterial community structures in the Amundsen Sea polynya. *Front. Microbiol.* 5:646 10.3389/fmicb.2014.00646 PMC4271704 25566197

[B9] Denef V. J. Kalnejais L. H. Mueller R. S. Wilmes P. Baker B. J. Thomas B. C. (2010). Proteogenomic basis for ecological divergence of closely related bacteria in natural acidophilic microbial communities. *Proc. Natl. Acad. Sci. U.S.A.* 107 2383–2390. 10.1073/pnas.0907041107 20133593 PMC2823883

[B10] Eren A. M. Borisy G. G. Huse S. M. Mark Welch J. L. (2014). Oligotyping analysis of the human oral microbiome. *Proc. Natl. Acad. Sci. U.S.A.* 111 E2875–E2884. 10.1073/pnas.1409644111 24965363 PMC4104879

[B11] Eren A. M. Maignien L. Sul W. J. Murphy L. G. Grim S. L. Morrison H. G. (2013). Oligotyping: differentiating between closely related microbial taxa using 16S rRNA gene data. *Methods Ecol. Evol.* 4 1111–1119. 10.1111/2041-210X.12114 PMC3864673 24358444

[B12] Eren A. M. Morrison H. G. Lescault P. J. Reveillaud J. Vineis J. H. Sogin M. L. (2015). Minimum entropy decomposition: unsupervised oligotyping for sensitive partitioning of high-throughput marker gene sequences. *ISME J.* 9 968–979. 10.1038/ismej.2014.195 25325381 PMC4817710

[B13] Eren A. M. Zozaya M. Taylor C. M. Dowd S. E. Martin D. H. Ferris M. J. (2011). Exploring the diversity of gardnerella vaginalis in the genitourinary tract microbiota of monogamous couples through subtle nucleotide variation. *PLoS ONE* 6:e26732 10.1371/journal.pone.0026732 PMC3201972 22046340

[B14] Fisher J. C. Murat Eren A. Green H. C. Shanks O. C. Morrison H. G. Vineis J. H. (2015). Comparison of sewage and animal fecal microbiomes by using oligotyping reveals potential human fecal indicators in multiple taxonomic groups. *Appl. Environ. Microbiol.* 81 7023–7033. 10.1128/AEM.01524-15 26231648 PMC4579428

[B15] Gevers D. Cohan F. M. Lawrence J. G. Spratt B. G. Coenye T. Feil E. J. (2005). Opinion: re-evaluating prokaryotic species. *Nat. Rev. Microbiol.* 3 733–739. 10.1038/nrmicro1236 16138101

[B16] Gobler C. J. Burkholder J. M. Davis T. W. Harke M. J. Stow C. A. Van de Waal D. B. (2016). The dual role of nitrogen supply in controlling the growth and toxicity of cyanobacterial blooms. *Harmful Algae* 54 87–97. 10.1016/j.hal.2016.01.010 28073483

[B17] Hahn M. W. Jezberová J. Koll U. Saueressig-Beck T. Schmidt J. (2016). Complete ecological isolation and cryptic diversity in *Polynucleobacter* bacteria not resolved by 16S rRNA gene sequences. *ISME J.* 10 1642–1655. 10.1038/ismej.2015.237 26943621 PMC4913878

[B18] Harke M. J. Davis T. W. Watson S. B. Gobler C. J. (2016a). Nutrient-controlled niche differentiation of western lake erie cyanobacterial populations revealed via metatranscriptomic surveys. *Environ. Sci. Technol.* 50 604–615. 10.1021/acs.est.5b03931 26654276

[B19] Harke M. J. Steffen M. M. Otten T. G. Wilhelm S. W. Wood S. A. Paerl H. W. (2016b). A review of the global ecology, genomics, and biogeography of the toxic cyanobacterium, *Microcystis* spp. *Harmful Algae* 54 4–20. 10.1016/j.hal.2015.12.007 28073480

[B20] Hunt D. E. David L. A. Gevers D. Preheim S. P. Alm E. J. Polz M. F. (2008). Resource partitioning and sympatric differentiation among closely related bacterioplankton. *Science* 320 1081–1085. 10.1126/science.1157890 18497299

[B21] Jaspers E. Overmann J. (2004). Ecological significance of microdiversity: identical 16S rRNA gene sequences can be found in bacteria with highly divergent genomes and ecophysiologies. *Appl. Environ. Microbiol.* 70 4831–4839. 10.1128/AEM.70.8.4831-4839.2004 15294821 PMC492463

[B22] Kim M. Oh H.-S. Park S.-C. Chun J. (2014). Towards a taxonomic coherence between average nucleotide identity and 16S rRNA gene sequence similarity for species demarcation of prokaryotes. *Int. J. Syst. Evol. Microbiol.* 64 346–351. 10.1099/ijs.0.059774-0 24505072

[B23] Kleindienst S. Grim S. Sogin M. Bracco A. Crespo-Medina M. Joye S. B. (2015). Diverse, rare microbial taxa responded to the Deepwater Horizon deep-sea hydrocarbon plume. *ISME J.* 10 1–16. 10.1038/ismej.2015.121 26230048 PMC4737931

[B24] Koeppel A. Perry E. B. Sikorski J. Krizanc D. Warner A. Ward D. M. (2008). Identifying the fundamental units of bacterial diversity: a paradigm shift to incorporate ecology into bacterial systematics. *Proc. Natl. Acad. Sci. U. S. A.* 105 2504–2509. 10.1073/pnas.0712205105 18272490 PMC2268166

[B25] Koeppel A. F. Wu M. (2013). Surprisingly extensive mixed phylogenetic and ecological signals among bacterial Operational Taxonomic Units. *Nucleic Acids Res.* 41 5175–5188. 10.1093/nar/gkt241 23571758 PMC3664822

[B26] Konstantinidis K. T. Tiedje J. M. (2005). Genomic insights that advance the species definition for prokaryotes. *Proc. Natl. Acad. Sci. U.S.A.* 102 2567–2572. 10.1073/pnas.0409727102 15701695 PMC549018

[B27] Kurmayer R. Blom J. F. Deng L. Pernthaler J. (2014). Integrating phylogeny, geographic niche partitioning and secondary metabolite synthesis in bloom-forming Planktothrix. *ISME J.* 9 909–921. 10.1038/ismej.2014.189 PMC4349496 25325384

[B28] Lauro F. M. McDougald D. Thomas T. Williams T. J. Egan S. Rice S. (2009). The genomic basis of trophic strategy in marine bacteria. *Proc. Natl. Acad. Sci. U.S.A.* 106 15527–15533. 10.1073/pnas.0903507106 19805210 PMC2739866

[B29] Lemaire V. Brusciotti S. van Gremberghe I. Vyverman W. Vanoverbeke J. De Meester L. (2012). Genotype × genotype interactions between the toxic cyanobacterium *Microcystis* and its grazer, the waterflea *Daphnia*. *Evol. Appl.* 5 168–182. 10.1111/j.1752-4571.2011.00225.x 25568039 PMC3353343

[B30] Maiden M. C. J. Jansen van Rensburg M. J. Bray J. E. Earle S. G. Ford S. A. Jolley K. A. (2013). MLST revisited: the gene-by-gene approach to bacterial genomics. *Nat. Rev. Microbiol.* 11 728–736. 10.1038/nrmicro3093 23979428 PMC3980634

[B31] Martiny A. C. Treseder K. Pusch G. (2013). Phylogenetic conservatism of functional traits in microorganisms. *ISME J.* 7 830–838. 10.1038/ismej.2012.160 23235290 PMC3603392

[B32] Menke S. Wasimuddin Meier M. Melzheimer J. Mfune J. K. E. Heinrich S., (2014). Oligotyping reveals differences between gut microbiomes of free-ranging sympatric Namibian carnivores (*Acinonyx jubatus*, *Canis mesomelas*) on a bacterial species-like level. *Front. Microbiol.* 5:526 10.3389/fmicb.2014.00526 PMC4196554 25352837

[B33] Newton R. J. McLellan S. L. (2015). A unique assemblage of cosmopolitan freshwater bacteria and higher community diversity differentiate an urbanized estuary from oligotrophic Lake Michigan. *Front. Microbiol.* 6:1028 10.3389/fmicb.2015.01028 PMC4586452 26483766

[B34] Ochman H. Elwyn S. Moran N. A. (1999). Calibrating bacterial evolution. *Proc. Natl. Acad. Sci. U.S.A.* 96 12638–12643. 10.1073/pnas.96.22.12638 10535975 PMC23026

[B35] O’Neil J. M. Davis T. W. Burford M. A. Gobler C. J. (2012). The rise of harmful cyanobacteria blooms: the potential roles of eutrophication and climate change. *Harmful Algae* 14 313–334. 10.1016/j.hal.2011.10.027

[B36] Otsuka S. Suda S. Li R. Watanabe M. Oyaizu H. Matsumoto S. (1999). Phylogenetic relationships between toxic and non-toxic strains of the genus *Microcystis* based on 16S to 23S internal transcribed spacer sequence. *FEMS Microbiol. Lett.* 172 15–21. 10.1111/j.1574-6968.1999.tb13443.x 10079523

[B37] Pobel D. Godon J. J. Humbert J. F. Robin J. (2012). High-frequency monitoring of the genetic diversity and the potential toxicity of a *Microcystis aeruginosa* bloom in a French shallow lake. *FEMS Microbiol. Ecol.* 79 132–141. 10.1111/j.1574-6941.2011.01203.x 22066470

[B38] Ramette A. Buttigieg P. (2014). The R package otu2ot for implementing the entropy decomposition of nucleotide variation in sequence data. *Front. Microbiol.* 5:601 10.3389/fmicb.2014.00601 PMC4231947 25452747

[B39] Schmidt V. T. Reveillaud J. Zettler E. Mincer T. J. Murphy L. Amaral-Zettler L. A. (2014). Oligotyping reveals community level habitat selection within the genus *Vibrio*. *Front. Microbiol.* 5:563 10.3389/fmicb.2014.00563 PMC4230168 25431569

[B40] Shapiro B. J. Polz M. F. (2014). Ordering microbial diversity into ecologically and genetically cohesive units. *Trends Microbiol.* 22 235–247. 10.1016/j.tim.2014.02.006 24630527 PMC4103024

