# Supplementary material for: Are Oligotypes Meaningful Ecological and Phylogenetic Units? A Case Study of Microcystis in Freshwater Lakes
Source: Front Microbiol. 2017 Mar 8;8:365. doi: 10.3389/fmicb.2017.00365 (PMC5341627; doi:10.3389/fmicb.2017.00365)
Supplement: Supplementary file 4 [file Supplementary_Code.ZIP › microcystis-oligotypes/R/mc-oligotype-analysis.pdf]

Microcystis Oligotype Analysis

Michelle Berry

December 2016

```
library(ggplot2)
library(dplyr)
library(grid)
library(gridExtra)
library(cowplot)
library(gtable)
library(tidyr)
library(pander)
library(ape)
theme_set(theme_bw())
```

Import Data

```
# Import oligotypes
mc_oligos <- read.csv(
  file = "data/mc-oligo-counts.txt",
  sep = "\t"
)

# Remove isolate data (keeping just erie samples)
erie_oligos <-
  mc_oligos %>%
    filter(grepl(pattern = "E0", x = samples))

sampdat <- read.csv("data/erie-sampledata.csv")
```

Format data

```
# Join sample data to oligotype data
oligos_join <-
  sampdat %>%
    left_join(erie_oligos, by = c("SampleID" = "samples"))

# Scale oligotype data by total reads from samples
oligos_scale <-
  oligos_join %>%
    mutate(CTT = CTT/ReadSums) %>%
    mutate(CTG = CTG/ReadSums) %>%
    mutate(CCG = CCG/ReadSums) %>%
    mutate(CTG_CCG_ratio = CTG/CCG)
```

```r
# Tidy oligotypes into one column
erie_oligo_df <-
  oligos_scale %>%
  select(-CCT, -TCG) %>%
  gather(key = Oligotype, value = count, CTG, CTT, CCG)


# Mean number of reads per sample
mean(erie_oligo_df$ReadSums)
```

```
## [1] 36926.25
```

```r
# Median number of reads per sample
hist(erie_oligo_df$ReadSums, main = "Histogram of sample read counts", xlab = "Read Sums")
```

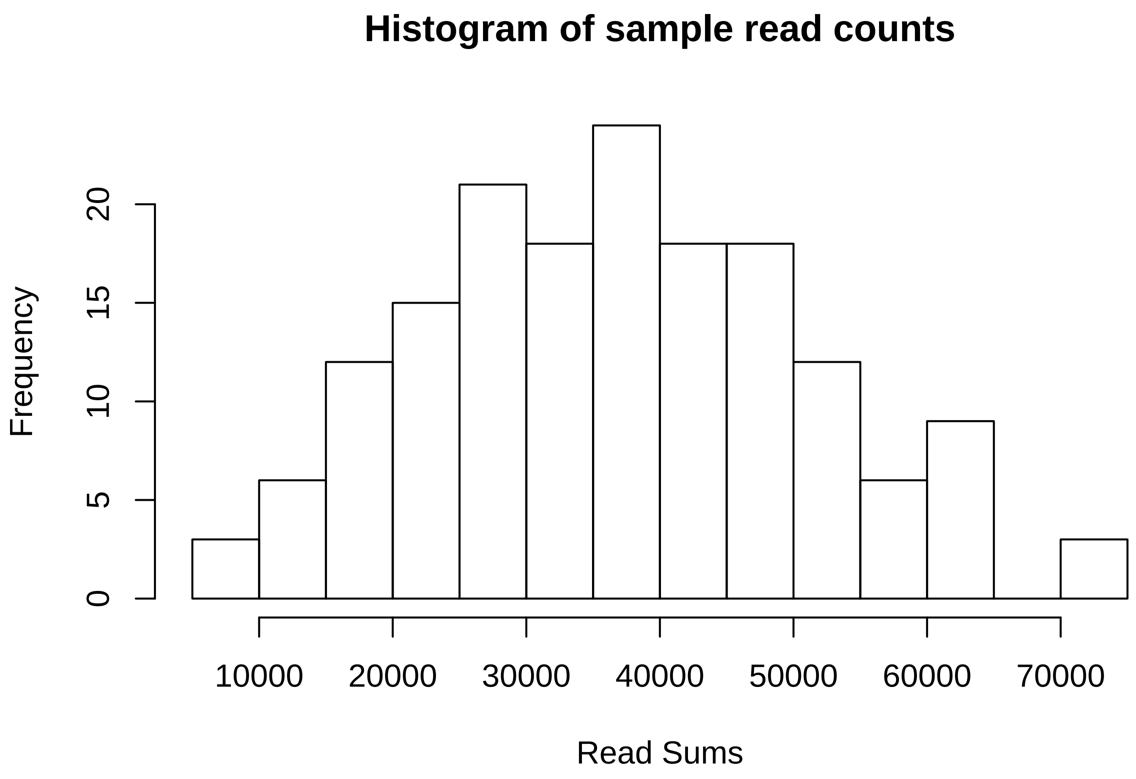

Plot data

```r
# Fixes the ordering of date factors
order_dates <- function(df){
  df$Date <- factor(df$Date,
    levels = c("6/16","6/30","7/8","7/14","7/21",
      "7/29","8/4","8/11","8/18","8/25","9/2","9/8","9/15",
      "9/23","9/29","10/6","10/15","10/20","10/27"))
  return(df)
}
```

```r
erie_oligo_df <- order_dates(erie_oligo_df)
erie_oligo_df$Oligotype <- factor(erie_oligo_df$Oligotype, levels = c("CCG", "CTT", "CTG"))


# Plot oligotypes over time
oligoplot <-
  ggplot(data = erie_oligo_df, aes(x = Date, y = count, fill = Oligotype)) +
    facet_grid(~Station) +
    geom_bar(stat = "identity") +
    scale_fill_manual(values = c("#b3002d", "#43a2ca", "#a6d854")) +
    scale_x_discrete(
      breaks = c("7/8", "8/4", "9/2", "10/6"),
      labels = c("Jul", "Aug", "Sep", "Oct"),
      drop = FALSE
    ) +
    theme(
      axis.text.x = element_text(angle = 45, vjust = 1, hjust = 1),
      axis.title.x = element_blank()
    ) +
    ylab("Relative Abundance \n (% of total community)")


# Plot toxin over time
toxinplot <-
  ggplot(data = erie_oligo_df, aes(x = Date, y = ParMC, group = Station)) +
    facet_grid(~Station) +
    geom_line(color = "black") +
    geom_point() +
  scale_x_discrete(
      breaks = c("7/8", "8/4", "9/2", "10/6"),
      labels = c("Jul", "Aug", "Sep", "Oct"),
      drop = FALSE
    ) +
  ylab("Particulate Microcystin-LR \n (ug/L)") +
  theme(
      axis.text.x = element_text(angle = 45, vjust = 1, hjust = 1),
      axis.title.x = element_blank(),
      plot.margin = unit(c(1, 0, 0, 0), "cm")
  )
```

```r
# grab grobs for PC plots
toxinGrob <- ggplotGrob(toxinplot)
oligoGrob <- ggplotGrob(oligoplot)

toxinGrobWider <- gtable_add_cols(toxinGrob, widths = oligoGrob$widths[11])
toxinGrobWider <- gtable_add_cols(toxinGrobWider, widths = oligoGrob$widths[11])

# rbind the two plots together
plot <- rbind(oligoGrob, toxinGrobWider, size = "first")


erie_plot <-
  ggdraw() +
```

```
    draw_plot(plot, x = 0.02, y = 0.02, width = 0.96, height = 0.94) +
    draw_plot_label(c("A", "B"), c(0, 0), c(1, .52), size = 20)

ggsave("../plots/raw-plots/erie-oligo-toxin-plot.pdf", plot = erie_plot, width = 10, height = 6)

erie_plot
```

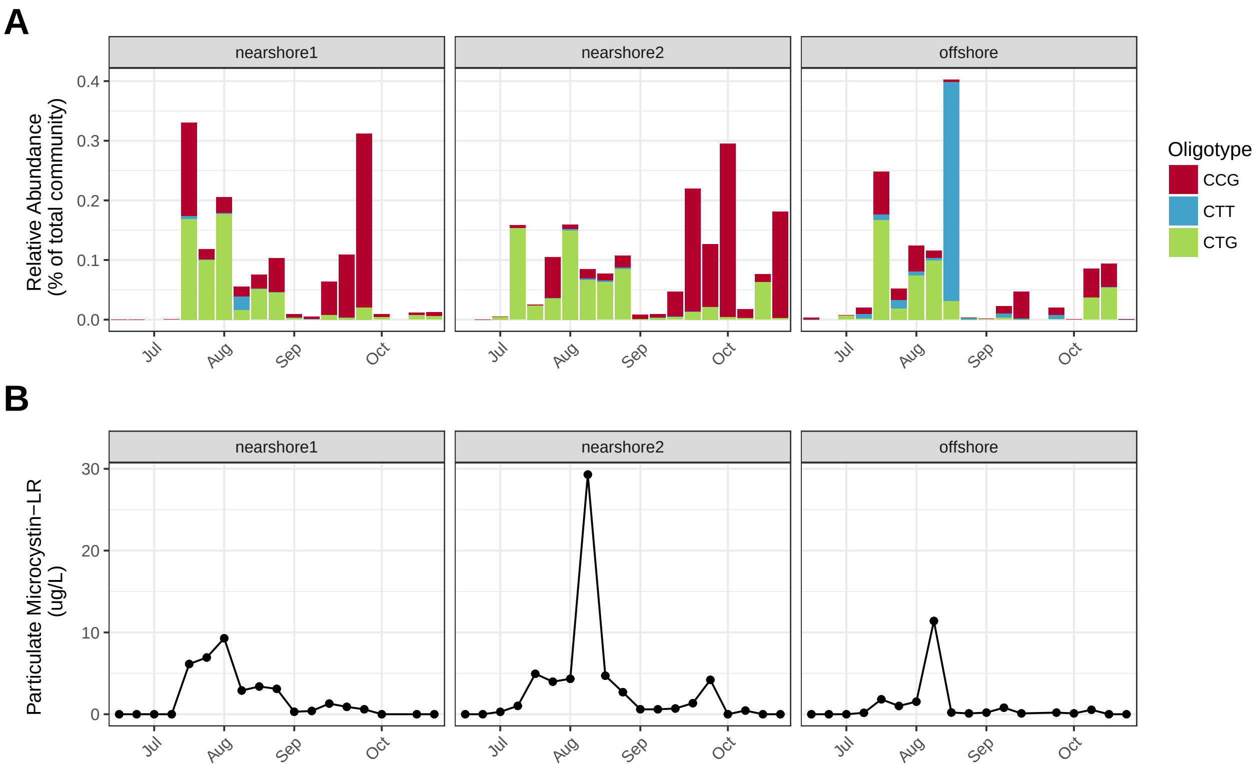

Statistics

Median Chla

```
erie_oligo_df %>%
  group_by(Station) %>%
  summarise(median(Chla)) %>%
  pander()
```

Station	median(Chla)
nearshore1	18.555
nearshore2	13.720
offshore	5.905

Median TP levels

```
erie_oligo_df %>%
  group_by(Station) %>%
  summarise(median(TP)) %>%
  pander()
```

Station	median(TP)
nearshore1	46.25
nearshore2	44.30
offshore	18.70

CTG, CCG, and toxicity

Filter out dates with no microcystis

```
# Ratio of CTG to CCG
ratio <-
  oligos_scale %>%
  filter((CTG + CCG + CTT) > 0)
```

Look at the median ratio of CTG to CCG in July and August

```
ratio %>%
  filter(Month %in% c( "July", "August")) %>%
  summarise(median(CTG_CCG_ratio)) %>%
  pander()
```

median(CTG_CCG_ratio)
4.339

Look at the median ratio of CTG to CCG in September and October

```
ratio %>%
  filter(Month %in% c("September", "October")) %>%
  summarise(median(CTG_CCG_ratio)) %>%
  pander()
```

median(CTG_CCG_ratio)
0.2347

Is there a significant correlation between CTG relative abundance and ParMC?

```
CTG <-
  erie_oligo_df %>%
  filter(Oligotype == "CTG")
```

```r
# Spearman's correlation
cor.test(CTG$count, CTG$ParMC, method = "spearman")
```

```
##
##  Spearman's rank correlation rho
##
## data:  CTG$count and CTG$ParMC
## S = 6918.7, p-value = 5.537e-09
## alternative hypothesis: true rho is not equal to 0
## sample estimates:
##       rho
## 0.7046571
```

```r
CTG_plot <- ggplot(CTG, aes(x = count, y = ParMC)) +
  geom_point() +
  ggtitle("CTG abundance vs Microcystin-LR toxin") +
  xlab("CTG relative abundance") +
  ylab("Microcystin-LR concentration \n (ug/L)")

ggsave("../plots/raw-plots/CTG_parmc.pdf", plot = CTG_plot, width = 7, height = 5)

CTG_plot
```

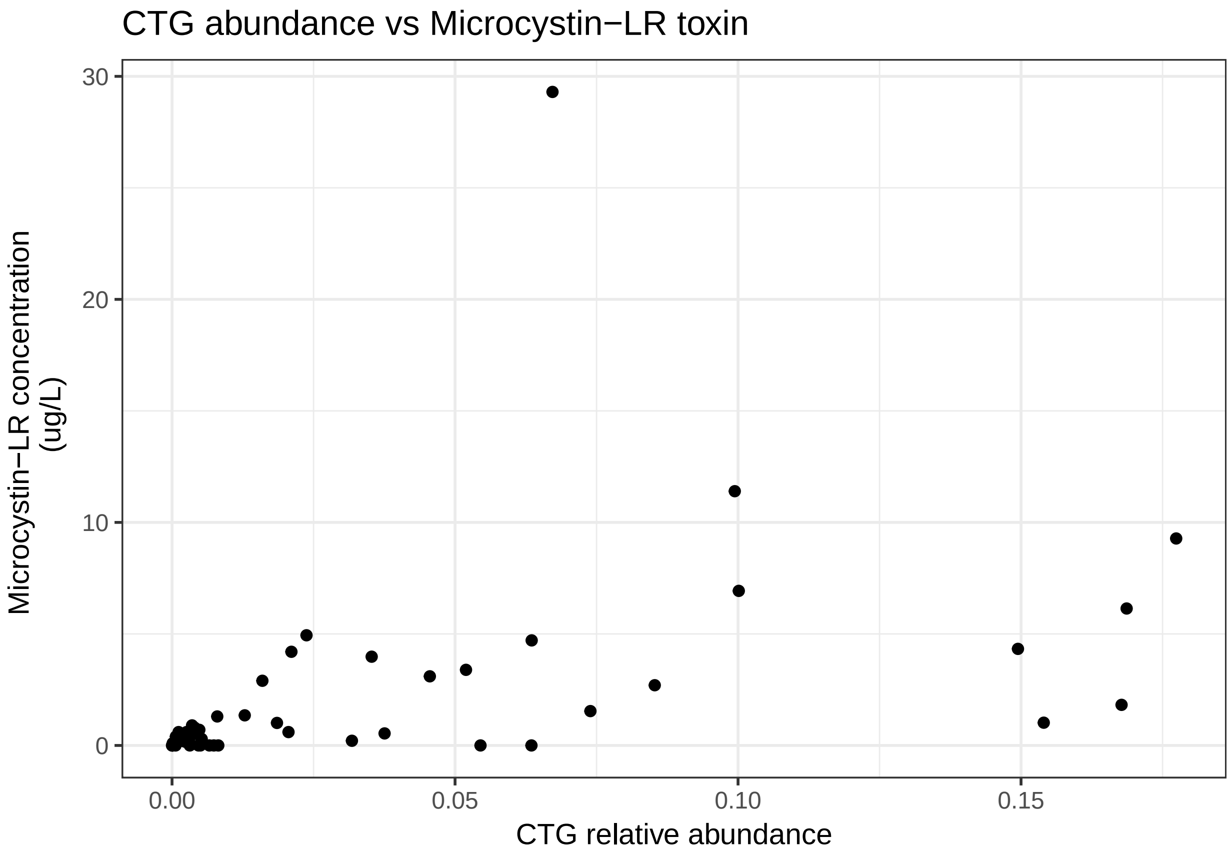

CTT and nutrient gradients

```
# Mean relative abundance
CTT <-
  erie_oligo_df %>%
    group_by(Shore) %>%
    filter(!is.na(count)) %>%
    filter(Oligotype == "CTT")

CTT %>%
  summarise(median(count), min(count), max(count)) %>%
  pander()
```

Shore	median(count)	min(count)	max(count)
nearshore	0.0002292592	0	0.02327592
offshore	0.0028197353	0	0.36665674

Distribution of nearshore and offshore abundance of CTT

```
CTT_plot <-
  ggplot(CTT, aes(x = count, group = Shore, fill = Shore)) +
    geom_density(alpha = .6, position = "identity") +
    scale_x_log10() +
    xlab("") +
    ggtitle("CTT relative abundance")

ggsave("../plots/raw-plots/CTT-distribution.pdf", plot = CTT_plot, width = 7, height = 5)

CTT_plot
```

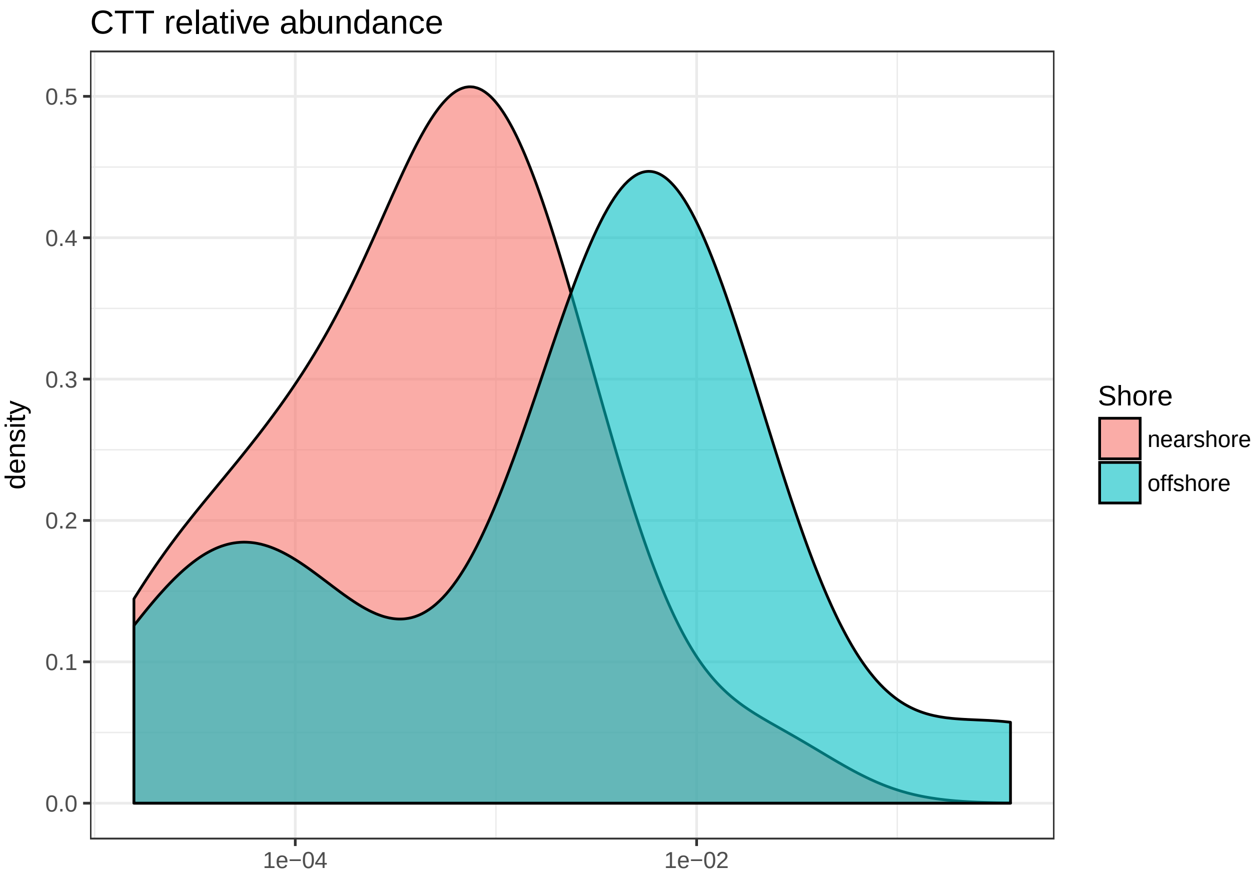

We will not calculate a statistic with a wilcoxon or permutational test, because our data violates the assumption of independence.

Supplementary figure 2

Here we read in the RaxML tree for the cultures and calculate their patristic distances

```
# Read in best raxml tree
rax_tree <- read.tree("data//RAxML_bestTree.bs100_mlst")

# Calculate patristic distances
patristic <- cophenetic(rax_tree)

# Remove the outgroup (NIES483) pulled from genbank
pat_dist <- as.dist(patristic[rownames(patristic) != "NIES483", colnames(patristic) != "NIES483"])
```

Here we calculate the hamming distance for the culture 16S-based oligotype sequence variants

```
# Filter to just culture samples
culture_oligos <-
  mc_oligos %>%
    mutate(sampID = substr(samples, 2, 10)) %>%
    filter(sampID %in% labels(pat_dist))
```

```r
# Most cultures have reads from multiple oligotype sequences.
# This can be caused by sequencing errors, contamination during library prep,
# or contamination in the cultures.
# This statement finds the consensus oligotype sequence for each culture i.e. the one with the maximum
cons_oligo <- apply(culture_oligos[,2:6], MARGIN = 1, function(x) {names(which.max(x))} )

# What percentage of the reads is the consensus sequence?
apply(culture_oligos[,2:6], MARGIN = 1, function(x) {max(x)/sum(x)} )
```

```
##  [1] 0.9822279 0.9984163 0.9951473 0.9962702 0.9962797 0.9971014 0.9975474
##  [8] 0.9424792 0.9736512 0.9919759 0.9849785 0.9961798 0.9947082 0.9944431
## [15] 0.9767895 0.9949935 0.9557522 0.9637263 0.9942591 0.9944651 0.9944523
## [22] 0.9956133 0.9795991 0.9982473 0.9979052 0.9930575 0.9943225 0.9962393
## [29] 0.9960454 0.9964202 0.9912559 0.9949810 0.9963345 0.9880606 0.9929515
## [36] 0.9974312 0.9958566 0.7696669 0.9656860 0.9954616 0.9919172 0.9973728
## [43] 0.9892725 0.9956514 0.9868641 0.9808088
```

With one exception (76.9%), the consensus sequence constitutes 94-100% of the reads.

```r
# Add consensus oligotype to data frame
culture_oligos$ConsOligo <- cons_oligo

# Order the samples the same as they are ordered in the tree
target <- labels(pat_dist)
culture_oligos_sorted <- culture_oligos[match(target, culture_oligos$sampID), ]


# Calculate hamming distance
ham_dist <- adist(culture_oligos_sorted$ConsOligo)
rownames(ham_dist) <- culture_oligos_sorted$sampID
colnames(ham_dist) <- culture_oligos_sorted$sampID
ham_dist <- as.dist(ham_dist)

# Sanity check
all.equal(labels(ham_dist), labels(pat_dist))
```

```
## [1] TRUE
```

Plot hamming distance vs patristic distance

```r
df <- data.frame(patDist = as.vector(pat_dist), hamDist = as.vector(ham_dist))

df$hamDist <- as.factor(df$hamDist)

hamdist_plot <-
  ggplot(df, aes(x = hamDist, y = patDist, color = hamDist)) +
    geom_boxplot() +
    ylab("Patristic Distance (RaxML tree)") +
    xlab("16S V4 hamming distance") +
    theme(legend.position = "none")
```

```r
ggsave("../plots/raw-plots/hamdist-vs-patristic-plot.pdf", plot = hamdist_plot, width = 7, height = 5)

hamdist_plot
```

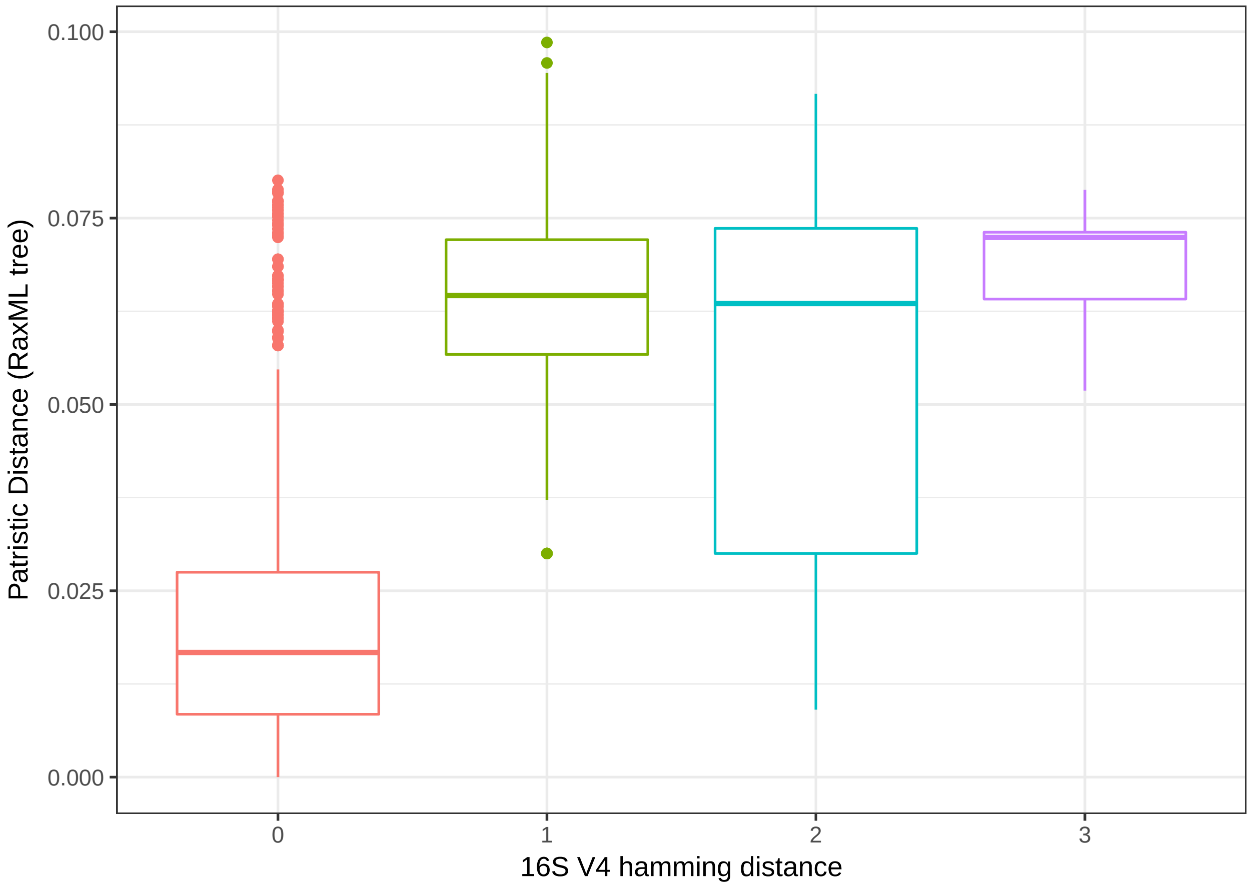


```r
sessionInfo()
```

```
## R version 3.3.0 (2016-05-03)
## Platform: x86_64-apple-darwin13.4.0 (64-bit)
## Running under: OS X 10.9.5 (Mavericks)
##
## locale:
## [1] en_US.UTF-8/en_US.UTF-8/en_US.UTF-8/C/en_US.UTF-8/en_US.UTF-8
##
## attached base packages:
## [1] grid      stats     graphics  grDevices utils     datasets  methods
## [8] base
##
## other attached packages:
## [1] ape_3.5         pander_0.6.0    tidyr_0.5.1     gtable_0.2.0
## [5] cowplot_0.6.2   gridExtra_2.2.1 dplyr_0.5.0     ggplot2_2.2.0
##
## loaded via a namespace (and not attached):
##  [1] Rcpp_0.12.5      knitr_1.13       magrittr_1.5     munsell_0.4.3
```

```
##  [5] lattice_0.20-33  colorspace_1.2-6 R6_2.1.2         stringr_1.0.0
##  [9] plyr_1.8.4       tools_3.3.0      nlme_3.1-128     DBI_0.4-1
## [13] htmltools_0.3.5  yaml_2.1.13      lazyeval_0.2.0   digest_0.6.9
## [17] assertthat_0.1   tibble_1.1       reshape2_1.4.1   formatR_1.4
## [21] evaluate_0.9     rmarkdown_1.0    labeling_0.3     stringi_1.1.1
## [25] scales_0.4.1
```
