# Supplementary material for: Are Oligotypes Meaningful Ecological and Phylogenetic Units? A Case Study of Microcystis in Freshwater Lakes
Source: Front Microbiol. 2017 Mar 8;8:365. doi: 10.3389/fmicb.2017.00365 (PMC5341627; doi:10.3389/fmicb.2017.00365)
Supplement: Supplementary file 5 [file Supplementary_Methods.PDF]

Supplementary Material

Are oligotypes meaningful ecological and phylogenetic units? A case study of *Microcystis* in freshwater lakes

Michelle A. Berry[1], Jeffrey D. White[2], Timothy W. Davis[3], Sunit Jain[4,§], Thomas H. Johengen[5], Gregory J. Dick[4], Orlando Sarnelle[6], and Vincent J. Denef[1*]

*** Correspondence:** Vincent Denef: vdenef@umich.edu

Materials and Methods

1.1 Lake Erie field sample collection

Samples were collected approximately weekly between mid-June and late October, 2014 from three stations (nearshore1, nearshore2, offshore) in the western basin of Lake Erie, which correspond to NOAA long-term monitoring sites WE12, WE2, WE4 respectively (NOAA-GLERL). Nearshore1 is closest to the water intake for the city of Toledo, nearshore2 is near the mouth of the Maumee River, and the offshore site is on the edge of typical bloom perimeter. Using a peristaltic pump, we collected a 20 L depth-integrated (0.5 m from surface - 1 m below bottom) water sample. Two liters of lake water was poured through a 100 µm Nitex mesh filter to collect the large colonial fraction. (Wildco, Inc, Yulee, FL). The retentate from the 100 µm mesh was backwashed into a falcon tube using altered BG-11 medium and RNAlater was added in a 2:1 ratio with the backwash. These samples were filtered onto a 47 mm diameter, 1 µm pore size Glass Fiber Filter (Millipore, Inc., Billerica, MA) with a syringe. After filtration, all filters were placed into 2 ml cryovials with 1 ml of RNAlater and frozen at -80 degrees C until extraction.

Particulate microcystins were extracted and analyzed using the the methods described in Davis et al. (2015) and reported in Cory et al. (2016). Phosphorus measurements were analyzed at the NOAA Great Lakes Environmental Research Laboratory using standard techniques (EPA 1979).

1.2 Inland lake sampling and culturing of laboratory strains

Water samples were collected via two pooled casts of an integrating tube sampler (12 m length × 2.5 cm i.d.) from the mixed layer of 14 lakes distributed across southern Michigan, between 5 July and 19 August, 2011, and again between 6-15 August, 2013 (Table S2). The lakes ranged widely in potential primary productivity as mean summer total phosphorus (TP; 7.9-196.8 µg L^{-1}, Table S2), determined using standard colorimetric techniques (molybdenum-blue method) and long path length spectrophotometry following persulfate digestion of organic matter (Murphy & Riley 1962; Menzel & Corwin 1965).

Microcystis was isolated under a dissecting microscope (16×, Leica MS5) by pipetting individual colonies through a series of six washes in sterile 0.5× WC-S growth medium within a well plate (Corning, Inc., Corning, NY), prior to being transferred into individual 20 mL tubes of growth

medium (White et al. 2011). Isolates were given unique designations identifying the originating lake, year, and strain number (e.g., BK11-02; Table S2). Once established, strains were maintained in 200 mL batch cultures of 0.5× WC-S medium at 23°C and ~80 µmol m^{-2} s^{-1} on a 12:12 h light:dark cycle, with an inoculum of culture transferred to fresh, sterile medium on a monthly basis. Subsamples of cultures for DNA analysis were filtered onto membrane filters, immediately frozen, and stored until extraction.

1.3 DNA extraction and sequencing

Filters were thawed at room temperature and for field samples, dipped into sterile PBS to remove RNAlater preservative. The filter was incubated in 100 µL Qiagen ATL tissue lysis buffer, 300 µL Qiagen AL lysis buffer, and 30 µL proteinase K for 1 hour at 56 degrees C on a rotisserie at maximum speed. Cells were lysed by vortexing at maximum speed for 10 minutes. Lysates were homogenized with the Qiashredder column, and DNA was purified from the filtrate using the DNeasy Blood and Tissue kit according to standard protocol (Qiagen, Hilden, Germany).

For 16S amplicon data, the V4 hypervariable region of the 16S rRNA gene was amplified from extracted DNA using primer set 515f/806r (Bergmann et al. 2011) in a polymerase chain reaction. Amplified DNA was sequenced using Illumina MiSeq v2 chemistry 2x250 (500 cycles) at the University of Michigan Medical School. RTA v1.17.28 and MCS v2.2.0 software were used to generate data.

For metagenomic data, extracted DNA was submitted to the University of Michigan sequencing core for Illumina HiSeq 100 cycle paired end sequencing (2 x 100 nt). Libraries with a target insert size of 500 nt were generated using the automated Apollo 324 library preparation system (Wafergen Biosystems, Fremont, CA). The contribution of each library to the pooled libraries sample that was sequenced on one lane of Illumina Hiseq was adjusted based on *Microcystis* relative abundance estimates from the 16S amplicon data, so as to get approximately equal coverage for *Microcystis* in all samples.

1.4 OTU and oligotyping analysis

We used mothur V 1.34.3 to perform quality control on raw sequence data, align reads, assign taxonomy, and cluster OTUs (Schloss et al. 2009). Sequence processing was performed according to the mothur standard operating procedure, accessed on March 13, 2016 (http://www.mothur.org/wiki/MiSeq_SOP). We assigned taxonomies to sequences using the Wang method (Wang et al. 2007) with an 80% bootstrap cutoff, using the Silva database V119 (Quast et al. 2013).

All sequences classified as *Microcystis* from both the Lake Erie dataset and inland lake isolates were selected for further analysis with oligotyping. Sequences were converted into the appropriate oligotyping format using the mothur2oligo script (https://github.com/DenefLab/MicrobeMiseq/tree/master/mothur2oligo, hash: 1bb42e6; June 3,

2016). We identified sites with nucleotide variation using the entropy-analysis command in the oligotyping pipeline (Eren et al. 2013). The entropy plot revealed three sites with considerable entropy (Figure S1), so we ran the oligotyping command with -c 3. We tested three different values (4, 10, 37) for the -M parameter, which serves to filter out noisy sequences by setting the minimum count of the most abundant unique sequence in an oligotype. . We tested these values, because the default setting is 4 (best for smaller datasets), the recommendation (Eren et al. 2013) for datasets with more than 10 samples or 10,000 reads per sample is the mean sample read count divided by 1,000 (36,926 for our dataset), and 10 served as an intermediate value. All values of M returned identical results with 5 final oligotypes. Entropy plots of the decomposed oligotypes were examined to make sure that oligotypes had converged on a single sequence.

R V3.2.2 (R Core Team 2015) and the ggplot2 package (Wickham 2009) were used to visualize *Microcystis* sequence variant patterns in Lake Erie samples. Using the cor.test command, a Spearman's rank correlation test was performed to assess the ordinal relationship between the relative abundance of the CTG variant and particulate microcystin-LR concentrations. Correlations between time-series can yield unstable or spurious results if the time-series are non-stationary i.e. have non-constant mean and variance over time. Neither detrending nor differencing resulted in stationary data, so we note the caveats of our analysis in the results. We report the median and maximum relative abundance of the CTT variant to assess differences between nearshore and offshore stations. We did not compute a wilcoxon-rank-sum test or equivalent test due to the violation of independent samples in our time-structured data.
All script, analysis, and data files are publically available at https://github.com/DenefLab/microcystis-oligotypes (hash 3c2bfb12810c557b78449322704c421430fdeda2, December 22, 2016)).

1.5 Genomic assembly and extraction MLST genes

Adapters trimming on raw reads was done using 'Scythe' (https://github.com/ucdavis-bioinformatics/scythe, hash 3ce3afe, September 24, 2015). Quality trimming was performed using 'Sickle' (Joshi & Fass 2011). Default parameters were used for both the tools. FastQC was used to assess the quality before and after the quality filtering. The bash script that combines these procedures that was used is located here: https://github.com/Geo-omics/scripts/blob/master/wrappers/Assembly/qc.sh (hash: cd95239; April 2, 2015). The filtered and trimmed sequencing reads were assembled using idba-ud as described previously (Anantharaman et al., 2014). We collected the gene sequences from the fully sequenced *Microcystis* strain NIES483 for five housekeeping genes (*pgi, gltX, ftsZ, glnA, gyrB*) previously used for MLST analysis (White et al. 2011), and a microcystin biosynthesis indicator gene (*mcyB*). Orthologs for these genes were searched for in the metagenomic data from each enrichment culture and extracted using a custom ruby script, which is available on this project's github page (https://github.com/DenefLab/microcystis-oligotypes, hash 3c2bfb12810c557b78449322704c421430fdeda2, December 22, 2016). Concatenated housekeeping gene sequences were aligned with MUSCLE using default parameters (Edgar et al., 2004) and a phylogenetic tree was reconstructed using RAxML 7.3.0 using standard parameters -T 10 (number of

threads) -x 777 (random seed number and activate rapid bootstrapping algorithm, which results in faster but qualitatively similar results than the standard method) -N 100 (number of alternative runs on distinct starting trees) (Stamatakis et al. 2005). While bootstrap support values were low for several of the branching points, in part due to high sequence similarity of the MLST genes within the isolate collection, several instances of polyphyletic oligotype groups were nonetheless evident.

Supplementary references:

Anantharaman, K., Duhaime, M.B., Breier, J.A., Wendt, K.A., Toner, B.M., and Dick, G.J., 2014. Sulfur oxidation genes in diverse deep-sea viruses. Science, 344(6185), 757-760.

Bergmann, G.T. et al., 2011. The under-recognized dominance of Verrucomicrobia in soil bacterial communities. Soil biology & biochemistry, 43(7), 1450–1455.

Cory, R. M., Davis, T. W., Dick, G. J., Johengen, T., Denef, V. J., Berry, M. A., et al. (2016). Seasonal Dynamics in Dissolved Organic Matter, Hydrogen Peroxide, and Cyanobacterial Blooms in Lake Erie. *Front. Mar. Sci.* 3, 54. doi:10.3389/fmars.2016.00054.

Davis, T.W., Bullerjahn, G.S., Tuttle, T., McKay, R.M., Watson, S.B., 2015. Effects of increasing nitrogen and phosphorus concentrations on the growth and toxicity of Planktothrix blooms in Sandusky Bay, Lake Erie. Environmental Science & Technology, 49(12): 7197 – 7207.

Edgar, R.C., 2004. MUSCLE: multiple sequence alignment with high accuracy and high throughput. Nucleic acids research, 32(5), 1792-1797.

Joshi N.A., Fass J.N.. (2011). Sickle: A sliding-window, adaptive, quality-based trimming tool for FastQ files (Version 1.33) [Software]. Available at https://github.com/najoshi/sickle.

Menzel, D.W. & Corwin, N., 1965. The measurement of total phosphorus in seawater based on the liberation of organically bound fractions by persulfate oxidation. Limnology and Oceanography, 10(2), 280–282.

Murphy, J. & Riley, J.P., 1962. A modified single solution method for the determination of phosphate in natural waters. Analytica Chimica Acta, 27, 31–36.

Quast, C. et al., 2013. The SILVA ribosomal RNA gene database project: Improved data processing and web-based tools. Nucleic Acids Research, 41(D1).

Schloss, P.D. et al., 2009. Introducing mothur: Open-Source, Platform-Independent, Community-Supported Software for Describing and Comparing Microbial Communities. Applied and Environmental Microbiology, 75(23), 7537–7541.

Stamatakis, A., Ludwig, T. & Meier, H., 2005. RAxML-III: a fast program for maximum likelihood-based inference of large phylogenetic trees. Bioinformatics, 21(4), 456–46310.

Wang, Q. et al., 2007. Naive Bayesian Classifier for Rapid Assignment of rRNA Sequences into the New Bacterial Taxonomy. Applied and Environmental Microbiology, 73(16), 5261–5267.

White, J., Kaul, R. & Knoll, L., 2011. Large variation in vulnerability to grazing within a population of the colonial phytoplankter, Microcystis aeruginosa. Limnology and Oceanography, 56(5), 1714–1724.
