# Supplementary material for: Are Oligotypes Meaningful Ecological and Phylogenetic Units? A Case Study of Microcystis in Freshwater Lakes
Source: Front Microbiol. 2017 Mar 8;8:365. doi: 10.3389/fmicb.2017.00365 (PMC5341627; doi:10.3389/fmicb.2017.00365)

Supplementary Figure 1. Shannon's entropy plot for aligned *Microcystis* sequences from Lake Erie samples and cultures. Using this plot, three nucleotide sites were selected for the oligotyping analysis. The three Lake Erie oligotypes differed at the second and third site, while the five culture oligotypes differed at all three sites.

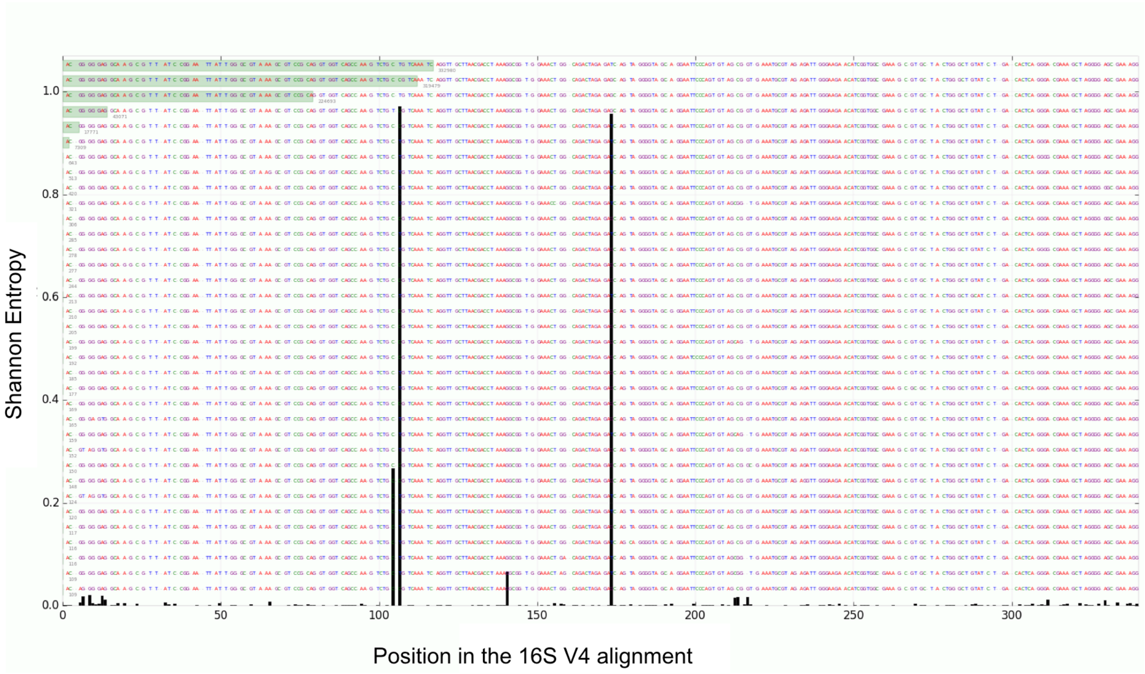

Supplement: Supplementary file 6 [file Image_1.PDF]
