# Supplementary material for: Are Oligotypes Meaningful Ecological and Phylogenetic Units? A Case Study of Microcystis in Freshwater Lakes
Source: Front Microbiol. 2017 Mar 8;8:365. doi: 10.3389/fmicb.2017.00365 (PMC5341627; doi:10.3389/fmicb.2017.00365)

Supplementary Figure 2. Hamming distance of the *Microcystis* culture oligotypes versus the patristic distances determined from a RaxML tree based on five concatenated housekeeping genes (Figure 2). Fine-scale variation (1-3 nucleotides) in the 16S rRNA V4 region did not correspond to patristic distances.

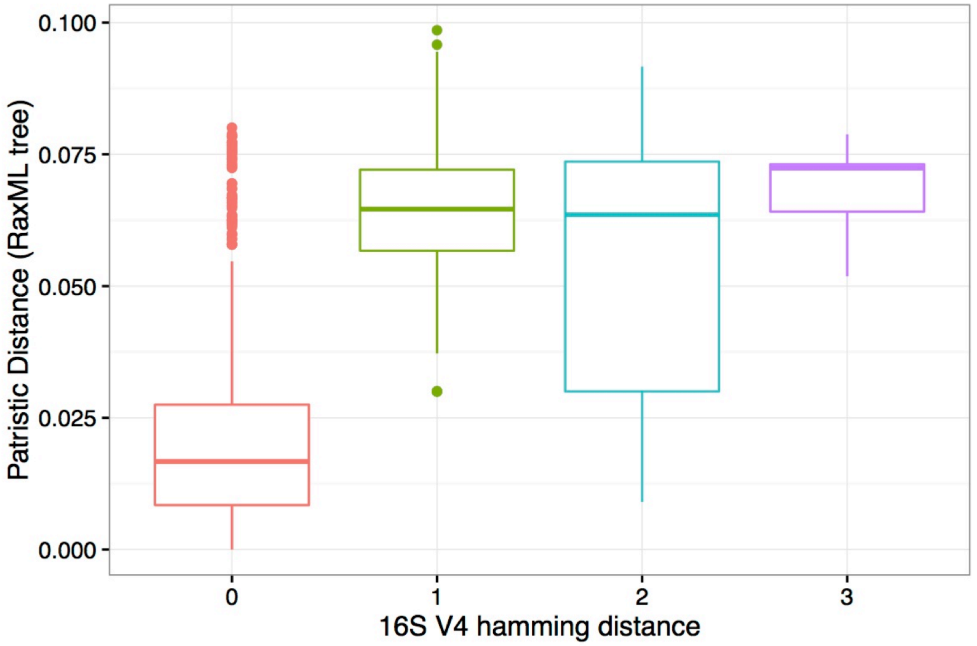

Supplement: Supplementary file 7 [file Image_2.PDF]
